# Aristarchus, Jun. in Explanation

**Published:** 1802-04

**Authors:** 


### *To the Editors of the Medical and Phyfical Journal.*

GENTLEMEN,

I Am happy to find that, to the honour of the practitioners in town, the tendency of my fecond query in your Journal (Vol. VII. p. 260) is not comprehended by you. It will neverthelefs, I am forry to add, be fully underftood by very many of your readers, who might not, a priori, be thought more likely to determine its allufion, did not their injured feelings bring it home to their underftandings in a manner equally forcible and unequivocal. I am not, however, of that party; and might rather, as an interefted being, applaud a practice which tends to increafe the number of my own fees, did I not feel that individual intereft fhould ever give place to public good. With this conviction ever paramount, I lend my feeble affiftance to the advancement of liberal fcience. Yet, while I affume the privilege of cenfuring the conduct or writings of others, I am well aware that I am no lefs open to *their* criticifm, to that of your *readers*, and *yourselves.* I feel that the language of my Query is too general and obfcure, and beg to fubmit the following as a much more comprehenfive and intelligible mode of putting a queftion, which will, I truft, be confidered as of fome importance.

Does it confift with the dignity, duty, or honefty of a Phyfician, to prefcribe in characters, or under denominations *unknown to the regular practitioners*; and which characters, or denominations, are underftood by the *Druggist* ALONE, who is deputed by fuch Phyfician as the *sole* compounder of his prefcriptions?

I do not find reafon to alter the remaining Queries. My objections are directed to the admiffion of *personal invective*, or *scurrile reflections*, againft the *writer*, and contend for the claims of the *subject matter* independent of the *man.*

I truly admire, though I may not have the power to imitate, the Attic falt of the Athenians; yet I do not admit *invective* or

*scurrility* as forming that falt in any fcientific difcuffion. It is true that my preceptor, Ariftophanes, was a mafter of all the acumen, diffufion, and graces of the Attic eloquence; and although in his *Nubes* he treated the ableft philofopher of his day with comic ridicule, who afterwards died a martyr to the envy and afperfion of his cotemporaries Anytus, Melites, and Lycon, yet I hope your Journal will not be poifoned like Socrates, or your correfpondents merit the comic ridicule of an Ariftophanes, or either meet the fate of the Anti-Socratic faction. Be this as it may, *I* fhall have the power of avoiding what I would confider as unbecoming, and fhall not deprive myfelf of the opportunity of collecting the *gold* from a work fo highly ufeful to the Profeffion, becaufe it may have an occafional alloy of *copper*. I am, &c.

*March* 4, 1802. ARISTARCHUS, Jun.

